# Postdocs should receive relocation benefits from the universities that hire them

**DOI:** 10.1128/aem.01483-24

**Published:** 2024-11-25

**Authors:** Zakee L. Sabree, Kayla Cross, James Gentry, Katie McAfee

**Affiliations:** 1 Department of Evolution, Ecology and Organismal Biology, The Ohio State University 170262 , Columbus, Ohio, USA; 2 Office of Postdoctoral Affairs, The Ohio State University 2647 , Columbus, Ohio, USA; 3 Department of Microbiology, The Ohio State University 215854 , Columbus, Ohio, USA; Michigan State University, East Lansing, Michigan, USA

**Keywords:** postdocs, relocation, moving benefits

## Abstract

Postdocs are essential to microbial science and STEM academic workforces but are underpaid and receive little-to-no relocation benefits. PhDs foregoing postdoctoral training for lucrative industry and government jobs exit the academic pipeline, which imperils current scholarship and the future professoriate. Relocation to postdoc jobs is expensive, especially for recent graduates and international scholars, but academia rarely provides support. Solving this short-term liquidity pressure can increase productivity, job satisfaction, and the likelihood they remain in academia.

## COMMENTARY

“Can I borrow your credit card?” Microbiologist Zakee Sabree put this question to his parents frequently during his transition into his first postdoctoral research position at the University of Arizona in Tucson. After earning his PhD in microbiology at the University of Wisconsin-Madison, he, like most postdocs, personally covered his relocation expenses, and it was uncommon for faculty PIs to provide relocation support. Nearly two decades since, PhDs moving to academic postdoc positions still are rarely offered relocation support benefits even though their labor is essential for basic and applied scholarship and innovations in academia. Most postdocs at the Ohio State University and other universities share the mix of joy of having earned their PhDs and the stress of figuring out how to afford moving to and living in a new town or city for the next step in their careers.

Employer-provided relocation support is common in industry, government, and nonprofit sectors, but it is rarely part of the initial offered hiring package for academic postdoctoral positions. The Ohio State University is like many research-intensive universities in that it does not offer this benefit to postdoc hires centrally nor does it require the hiring faculty member to provide it as standard practice. By contrast, Abbott, a global biotech firm, and Battelle Memorial Institute, an applied science and technology development firm, both recruit many microbial science and STEM PhDs to their Columbus, OH, campuses and typically offer relocation benefits and moving assistance to their new hires. Relocation support is “not just about moving belongings but transitioning your life…[r]eal life decisions are centered around this,” said Tara Schwetz, Director of the National Institutes of Health’s Division of Program Coordination, Planning, and Strategic Initiatives. Schwetz cochaired the NIH Advisory Committee to the Director Working Group on Re-envisioning NIH-Supported Postdoctoral Training, which sought to optimize the effectiveness of postdoctoral training and professional development to improve the postdoctoral experience for both the postdoc and the broader biomedical ecosystem. Since 2002, the number of newly minted PhDs that have chosen careers outside of academia continues to increase by a rate of 1% per year, based on the 2022 National Science Foundation NCSES Survey of Earned Doctorates (1). The percentage of recent PhD graduates pursuing postdoctoral training in 2002 was 75.3%, and by 2022, this rate decreased to 59.5%. The NIH working group final report (2) cited surveys conducted by the *Nature* journal (3, 4) and the National Postdoctoral Association (5) that paint a similarly bleak picture: less than half of postdocs felt positively about their job prospects, most report mental health concerns as reasons for considering leaving their fields and low salary and lack of healthy workplace culture as strong negative impacts on their lives (2–5). Since postdoc training is all but required for most tenure-track positions, continued leakage of PhDs from the academic pipeline imperils the future professoriate and hampers current academic research and scholarly productivity.

When virologist Marion Urvoy accepted a postdoc position at Ohio State, her faculty mentor was able to provide limited relocation support to assist with her move to Columbus, Ohio, from France, but the associated costs were still a concern. “I am not sure I would have made the move immediately after my PhD if I didn’t have personal savings available. I feel like I might have started a postdoc somewhere more affordable in France, my home country, or in Europe.” Relocation costs can be thousands of dollars to cover air and/or ground transportation, hotels, short-term rentals, security deposits for long-term rentals, dependent care, meals and incidentals, hiring movers, etc., and these costs can rise sharply for international postdocs who may have additional expenses related to international travel, obtaining work visas, and limited access to banking in the USA. PhDs in the USA moving to postdoc positions within the USA are also burdened with many of the same financial pressures and expectations to cover their own relocation costs.

To understand if and how US universities provided postdoc relocation support, the Ohio State University Office of Postdoctoral Affairs (OPA) reviewed the publicly available human resource policies governing moving expenses or relocation support policies for 49 research-intensive institutions, which included all Ivy League, Big10, and Big10-affiliated universities, and looked for language therein that described (i) if postdocs were eligible for relocation or moving expenses and (ii) how that support was to be funded (i.e., from the faculty advisor, departmental or central hiring unit). Additionally, OPA directly contacted university postdoc support and HR offices via email and telephone with the same questions when the policies did not explicitly mention postdocs, and a minimum of 2 weeks was allowed for response. We found that only the Perdue University and OSU policies stated that postdocs were eligible recipients of relocation support, and an additional 11 universities reported through their representatives that postdocs could receive relocation support (Table 1). In all cases, the costs of providing relocation support when allowed were to be borne by the faculty mentor or hiring unit (Table 1).

**TABLE 1:** Few relocation support policies at research-intensive universities provide clear guidance regarding postdocs

University	Link(s) to university relocation policy^*a*^	Are postdocs mentioned in the institution’s relocation policy?	Are postdocs eligible for relocation support and who funds it?^*b*^	What is the maximum for postdocs?
Brown University	https://policy.brown.edu/policy/moving-expenses	Y	N	N.A.^*c*^
Columbia University	https://worklife.columbia.edu/content/relocation	N	Y; hiring unit funds the support	$1,500
Cornell University	https://finance.cornell.edu/procurement/buyers/commodities/moving/household-quick-guide https://finance.cornell.edu/tax/fordepartments/moving	N	Y; hiring unit funds the support	N.A.
Duke University	https://finance.duke.edu/travel/reimbursement/moving https://careers.dukehealth.org/relocation	N	Y; hiring unit or faculty mentor funds support	Up to $2,000
Harvard University	https://oc.finance.harvard.edu/how-to/tax-services/moving-recruiting-and-related-expenses	N	N.A.	N.A.
Massachusetts Institute of Technology	https://vpf.mit.edu/expense-your-relocation	N	Y; faculty mentor funds support	N.A.
Princeton University	https://finance.princeton.edu/buying-paying/pay-and-reimburse-individuals/pay-moving-or-relocation-expenses	N	N.A.	N.A.
Stanford University	https://adminguide.stanford.edu/chapters/human-resources/staff-employment-policies/relocation-faculty-and-staff	N	Y; hiring unit funds the support	No maximum
University of Pennsylvania	https://www.finance.upenn.edu/policy/2324-reimbursement-of-moving-expenses/	N	N.A.	N.A.
Yale University	https://your.yale.edu/policies-procedures/policies/3510-faculty-staff-relocation-expenses	N	N.A.	N.A.
Arizona State University	https://cfo.asu.edu/az-resources	N	N.A.	N.A.
Auburn University	https://www.auburn.edu/administration/human_resources/procedures/documents/employee-transition.pdf	N	N.A.	N.A.
University of Alabama at Birmingham	https://www.uab.edu/financialaffairs/traveling/employee/moving-relocation-allowance	N	N.A.	N.A.
Boston University	https://www.bu.edu/travelservices/files/2018/05/Travel-Policy-Final-May2018.pdf	N	N.A.	N.A.
Georgia Institute of Technology	https://policylibrary.gatech.edu/academic-affairs/relocation-assistance	N	Y; faculty mentor funds support	N.A.
Virginia Tech	https://www.controller.vt.edu/content/dam/controller_vt_edu/procedures/travel/2034B.pdf	N	N.A.	N.A.
Texas A&M University	https://rules-saps.tamu.edu/PDFs/31.01.99.M0.03.pdf	N	N.A.	N.A.
Stony Brook University	https://www.stonybrook.edu/commcms/procurement/employees/reimbursement_of_relocation.php	N	N.A.	N.A.
UC - San Diego	https://www.sandiego.edu/provost/faculty-affairs/recruitment-and-relocation.php#:~:text=At%20the%20discretion%20of%20each,moving%20expense%20allowance%20is%20%245%2C000.	N	N.A.	N.A.
UC - San Francisco	https://supplychain.ucsf.edu/employee-moving-and-relocation	N	N.A.	N.A.
UC - Berkeley	https://controller.berkeley.edu/financial-operations/accounts-payable/moving-and-relocation-expenses	N	N.A.	N.A.
University of Colorado -Boulder	https://www.cu.edu/employee-services/payroll/moving	N	N.A.	N.A.
University of Colorado -Denver	https://www.cu.edu/employee-services/payroll/moving	N	N.A.	N.A.
University of Illinois - Chicago	https://hr.uic.edu/news-stories/relocation-assistance-information-alert/#:~:text=The%20new%20policy%20allows%20the,Affairs%2C%20Provost%20or%20their%20designee.	N	Y; faculty mentor or hiring unit funds support	Up to $15,000
University of Illinois - Urbana-Champaign	https://www.uiucpurchasing.illinois.edu/moving-relocation/	N	N.A.	N.A.
UNC - Chapel Hill	https://policies.unc.edu/TDClient/2833/Portal/KB/ArticleDet?ID=131454#:~:text=Policy%20Statement,order%20to%20accept%20the%20position.	N	Y; hiring unit funds the support	10% base salary
University of Pittsburgh	https://www.policy.pitt.edu/sites/default/files/Relocation_Policy-Public_Comment.pdf	N	N.A.	N.A.
Washington University of St. Louis	https://financialservices.wustl.edu/wp-content/uploads/2018/12/Relocation-Expense-Guide-rev-2019.pdf	N	Y; faculty mentor funds support	No maximum
Indiana University Bloomington	https://policies.iu.edu/policies/fin-acc-310-moving-expenses/archived-07202015-06042016.html	N	N.A.	N.A.
University of Maryland, College Park	https://purchase.umd.edu/services/physical-distribution/household-goods-and-laboratory-moves	N	Y; hiring unit funds the support	N.A.
University of Michigan	https://spg.umich.edu/sites/default/files/policies/201x68x00_11-30-2023.pdf	N	N.A.	N.A.
Michigan State University	https://ctlr.msu.edu/payroll/moving-expense-info	N	N.A.	N.A.
Pennsylvania State University	https://policy.psu.edu/policies/hr104	N	Y; hiring unit funds the support	Up to $15,000 + 1 month salary
Rutgers University - New Brunswick	https://procurementservices.rutgers.edu/resources/how-to/expense/process-employee-relocation-reimbursement	N	N	N.A.
University of Illinois - Urbana-Champaign	https://www.uiucpurchasing.illinois.edu/moving-relocation/	N	N.A.	N.A.
University of Iowa	https://hr.uiowa.edu/pay/compensation-classification/professional-scientific-compensation/moving-relocation-assistance	N	N.A.	N.A.
University of Minnesota, Twin Cities	https://hr.umn.edu/Jobs/Applicant-Center/Relocation-Assistance-Program#:~:text=Moving,and%20subject%20to%20tax%20withholding.	N	N.A.	N.A.
University of Nebraska - Lincoln	https://nebraska.edu/-/media/unca/docs/offices-and-policies/policies/policies/bf-01-moving-relocation-policy.pdf	N	N	N.A.
Northwestern University	https://hr.northwestern.edu/benefits/discounts-transit/relocation/housing-moving/moving-expense-reimbursement.html	N	N.A.	N.A.
Purdue University	https://www.purdue.edu/policies/business-finance/iia2.html	Y	Y; hiring unit funds the support	Up to $5,000
University of Wisconsin - Madison	https://policy.wisc.edu/library/UW-3079	N	N.A.	N.A.
University of Southern California	https://policy.usc.edu/recruitment-and-relocation-expenditures/#:~:text=All%20or%20part%20of%20new,university%20attract%20highly%20qualified%20candidates.	N	N.A.	N.A.
University of California – Los Angeles	https://apo.ucla.edu/faculty-resources-new-faculty/relocation	N	N.A.	N.A.
University of Oregon	https://ba.uoregon.edu/moving/relocation-expenses-department-instructions	N	N.A.	N.A.
University of Washington	https://hr.uw.edu/talent/hiring-process/employee-relocation-incentive/	N	N.A.	N.A.
Johns Hopkins University	https://hrpayroll.ssc.jhu.edu/wp-content/uploads/sites/14/moving_allowance.pdf	N	N.A.	N.A.
University of Notre Dame	https://policy.nd.edu/assets/185251/moving_expense.doc	N	N.A.	N.A.
University of Chicago	https://lbc.ssd.uchicago.edu/expenses-and-reimbursements/account-reconciliations-and-policies/relocation-reimbursements/	N	N.A.	N.A.
Ohio State University	https://hr.osu.edu/wp-content/uploads/policy230.pdf	Y	Y; hiring unit or fauclty mentor funds the support	Up to $5,000

^*a*^ Links active as of 4 September 2024.

^*b*^ University postdoc support or HR administrators responded to questions about allowable postdoc relocation support when contacted by email or telephone.

^*c*^ N.A., information was not available either from the published policies or from contacted HR administrators.

In this context, OPA implemented a postdoc relocation support program that could provide support independent of faculty mentor or hiring units, making it unique among its institutional peers. With generous funding from the W. K. Kellogg Foundation, OPA was enabled to provide monetary awards to postdocs being recruited or recently hired by faculty at Ohio State within the previous 12 months. Under the moniker “the Ohio State University W.K. Kellogg Foundation Postdoctoral Recruitment Onboarding Supplement” or “OK-PROS” program (6), we sought to determine if providing institution-level relocation support would positively impact postdoc recruitment, occupational success, and sentiments/perceptions of Ohio State. Beginning in late 2022, 51 postdocs each received $5,000 awards that they could use to cover or reimburse any costs associated with moving to Columbus and provide liquidity until their first paycheck. Additionally, postdocs could use remaining funds to support other career development activities as they established themselves and onboarded to the university. Under this pilot project, Ohio State was the first and only institution in the BigTen Academic Alliance to offer relocation support directly to postdocs. Awardees were 62% women, 37% non-White, and 40% US citizens, based on self-reported demographics. Furthermore, award recipients were employed in microbiology, evolutionary biology, biomedical and other STEM departments, as well as arts and humanities departments. Surveys were administered to OK-PROS postdoc award recipients and their faculty advisors to capture the impacts of the award on postdoc relocation experience, productivity, and perceptions of the university. >70% of postdoc awardee respondents (*n* = 45), primary uses of the support were relocation expenses (e.g., shipping personal items, paying for dependent care, air travel, lodging, and meals) and short-term liquidity until the first paycheck. “Postdocs are often financially and personally stretched. Especially for persons with less access to financial resources and international postdocs,” said Schwetz. Aquatic biogeochemist Ashlynn Boedecker used the award to cover debt associated with relocation. “It was very helpful to pay off relocation expenses, since I had to move ~1,200 miles away on a graduate student stipend. Rent in Columbus was almost my entire last paycheck from my PhD, so it was essential to be able to eat and live.” Similarly, behavioral health scientist Andrea Garcia said the OK-PROS award helped her to “manage childcare expenses, especially considering the higher cost of living in Columbus compared to Omaha, Nebraska. This support facilitated a seamless transition, enabling me to shift my focus away from financial concerns and more towards orienting myself in my new position.”

Of 35 OK-PROS awardee faculty advisor respondents, 77% reported that they felt relocation support provided crucial resources for a smooth start, improved the overall training experience, demonstrated institutional commitment and support, and reduced financial burden. Most faculty felt postdocs should receive relocation support, but they often did not provide it themselves because they were confused by vague university policies on how to provide relocation support to postdocs, felt constrained by grant budgets, or were uncertain whether relocation was an allowable expense on their grants. Additionally, 71% of faculty reported that they did not receive relocation support when they were postdocs, which highlights the intergenerational normativity of not providing postdocs with relocation support. Among federal agencies, the National Science Foundation and National Institutes of Health are both major funders of research in the biomedical and microbial sciences and STEM disciplines, and relocation support to project staff, including postdocs, are allowable expenses for NSF core program and NIH R01 grants, unless specifically indicated otherwise. Schwetz acknowledged that clearer advice to faculty and universities is needed about how postdoc relocation support can be provided from NIH grants. OPA’s survey of research-intensive universities concurs with the need for clear, easily accessible guidance on postdocs receiving relocation support (Table 1). The absence of this information could discourage faculty from requesting these funds as part of their grantmaking activities or mislead HR representatives to provide inaccurate guidance to faculty.

Nearly all faculty respondents reported that universities should directly provide a relocation benefit to postdocs and that doing so would improve their opinion of Ohio State and signal strong institutional support to both current and potential faculty hires. Macarius Donneyong, Associate Professor of Pharmacy and Public Health at Ohio State, observed that “defraying the costs for relocation to Columbus was a direct benefit of the award” to his postdoc, Ximena Oyarzún-González. “As a result, she was able to settle in relatively quickly and helped me co-author two publications,” said Donneyong. Schwetz, echoed Donneyong’s conclusion, stating that relocation support “could play a critical role in ameliorating the transition and easing the burden…empowering researchers by removing various barriers and helping to set the [principal investigator’s] lab up for success.”

Interviews with several members of the recent NIH postdoc experience working group cochaired by Schwetz yielded a consensus that institution-level relocation benefits would bolster efforts to recruit and retain PhDs in the academic pipeline. “It’s not a lot of money, but it is a lot of money to [postdocs],” said Donna Ginther, Roy A. Roberts & Regents Distinguished Professor of Economics and Director of the University of Kansas Institute for Policy & Social Research, regarding the relative value of relocation support and its impact on the postdoc experience. “Relocation support shows care,” said Rashada Alexander, Director of the Science & Technology Policy Fellowship Program at the American Association for the Advancement of Science. “This kind of investment is good…people are putting their energy and commitment into research and it makes sense that the institution should be committing to them also,” said Alexander. The National Postdoctoral Association has been a strong advocate for relocation benefits for postdoctoral scholars, stating that “institutions should consider providing [moving expenses] allowances to all incoming postdocs regardless of financial need to ensure greater parity” (7). NPA Executive Director Thomas Kimbis expressed strong support for an institution-level relocation benefit program as modeled by the OK-PROS award program, stating that he “would love to see this pilot program spread to other institutions.” Moving expense allowances were included in the NPA’s 2024 “Recommended Policies and Practices” to improve the postdoctoral training experience and environment (7).

Faculty who want to use relocation benefits as a recruitment tool, but who have budget constraints, could request that these funds be included in proposed start-up packages when negotiating for new roles or retention packages. Leaders of hiring units could also work with their institution’s postdoctoral support units to advocate for policy changes at college and institutional levels that make relocation benefits standard practice for future postdoc hires. Furthermore, universities should provide clear, easily accessible guidance to faculty, hiring units, and HR administrators on how relocation support can be provided to postdocs. Institution-level relocation benefits are a nominal expenditure for universities and are already standard practice for faculty and senior administration recruitment practices. Standardizing the inclusion of postdocs into these policies would positively impact recruitment and retention of early career scientists and scholars in academia.
